# Thermally damaged porcine skin is not a surrogate mechanical model of human skin

**DOI:** 10.1038/s41598-022-08551-z

**Published:** 2022-03-16

**Authors:** Samara Gallagher, Uwe Kruger, Kartik Josyula, Rahul, Alex Gong, Agnes Song, Robert Sweet, Basiel Makled, Conner Parsey, Jack Norfleet, Suvranu De

**Affiliations:** 1 Department of Mechanical, Aerospace, and Nuclear Engineering, Rensselaer Polytechnic Institute, Troy, NY USA; 2 Center for Modeling, Simulation, and Imaging in Medicine, Rensselaer Polytechnic Institute, Troy, NY USA; 3 Department of Biomedical Engineering, Rensselaer Polytechnic Institute, Troy, NY USA; 4 Center for Research in Education and Simulation Technologies, University of Washington, Seattle, WA USA; 5 U.S. Army Combat Capabilities Development Command-Soldier Center, Simulation and Training Technology Center, Orlando, FL USA

**Keywords:** Biomechanics, Mechanical properties

## Abstract

Porcine skin is considered a de facto surrogate for human skin. However, this study shows that the mechanical characteristics of full thickness burned human skin are different from those of porcine skin. The study relies on five mechanical properties obtained from uniaxial tensile tests at loading rates relevant to surgery: two parameters of the Veronda-Westmann hyperelastic material model, ultimate tensile stress, ultimate tensile strain, and toughness of the skin samples. Univariate statistical analyses show that human and porcine skin properties are dissimilar (p < 0.01) for each loading rate. Multivariate classification involving the five mechanical properties using logistic regression can successfully separate the two skin types with a classification accuracy exceeding 95% for each loading rate individually as well as combined. The findings of this study are expected to guide the development of effective training protocols and high-fidelity simulators to train burn care providers.

## Introduction

Porcine skin is considered to be anatomically and physiologically similar to human skin^1,2^. It has been used as a surrogate for human skin for evaluating mechanical characteristics under various thermomechanical loading conditions^3–5^. However, the mechanical properties of skin tissue change under thermal injury^6,7^. Moreover, there is no study on comparing the mechanical properties between full thickness burned human and porcine skin tissues. Such studies are important, however, to develop high-fidelity simulators and training protocols to train burn care providers. This is the first paper contrasting the mechanical properties of the full thickness burned human and porcine skin tissues to quantify the statistical similarity between the two.

Porcine skin is the closest animal surrogate of human skin tissue, as they have similar dermal-epidermal thickness ratios. The collagen and elastic fibers present in the dermis layer of the skin provide strength and elasticity to the skin^1^. Although the dermal collagen in porcine skin tissue is biochemically similar to that in human skin^2^, the elastic fiber content in human skin tissue is higher than that in porcine^8^. The porcine and human skins are tightly attached to the subcutaneous connective tissue, as opposed to rodent skin which is loosely attached^1^. Burn wounds in pigs and humans heal by re-epithelialization instead of wound contraction, which is observed in rodents. Pig and human skin have similar pigmentation, protein, and lipid composition, as well as similar sizes, orientations, and distribution of blood vessels^1^.

The mechanical behavior of human skin is considered similar to porcine skin tissue^3,4,9^. However, the tangent modulus of human and porcine skin tissues obtained from ex vivo uniaxial tensile tests, are reported to differ by two orders of magnitude^10,11^. A similar difference in the elastic modulus of the two types of skin tissues obtained for anatomically similar locations has been observed^11,12^. The stress–strain data of porcine skin tissue are found to be close to that of human skin tissue in some studies^3,9^ and significantly different in other study^9^. A comparison of the tangent modulus of human and porcine skin tissues from various experiments reported in the literature indicates that human skin is different from porcine skin in many cases^13^.

An analysis of variance revealed that the out-of-plane Young’s modulus of the stratum corneum of human skin tissue is greater than that of porcine. However, the out-of-plane Young’s modulus of viable epidermis/dermis composite samples and full thickness of human skin are less than those of porcine. It has been suggested that the stratum corneum of human and porcine skin might be different in its interactions with moisture and moisture absorption^14^. Interestingly, the Young’s modulus of the stratum corneum of the human and porcine skin tissues are ~ 111 MPa after freezing to − 80 °C and at 100% relative humidity. The change in temperature due to freezing is found to affect the Young’s modulus of stratum corneum and full thickness of human and porcine skin tissues in the exact opposite manner^15^. However, these comparisons are limited to unburned skin tissues. Further, there is no study that performed a statistical comparison of the mechanical properties of burned human and porcine skin tissues.

The hypertrophic scar tissue from a burn injury is stiffer than normal human skin tissue^7,16^. Similarly, the mechanical properties of burned and unburned porcine skin tissues are found to be significantly different^17^. Porcine skin is known to soften with an increase in temperature along with a change in the transition region of the typical J-curve response of skin tissues. This softening behavior is more prominent at higher strain values^4^. Hardening of the porcine skin is also observed with an increase in strain rate^4^. Hence, it is important to quantify whether full thickness burned porcine skin can be considered as a surrogate for full thickness burned human skin at various strain rates.

To the best of our knowledge, this is the first paper to study statistical similarity between the mechanical properties of full thickness burned ex vivo porcine and human skin tissues. Five mechanical properties, namely, ultimate tensile stress, ultimate tensile strain, toughness, and two parameters of the Veronda-Westmann hyperelastic material model^18^, are calculated from stress–strain data collected from uniaxial tensile tests on full thickness burned human and porcine skin tissues. The uniaxial tests are performed at three loading rates: 0.3 mm/s, 2.0 mm/s, and 8.0 mm/s. The loading rate of 0.3 mm/s corresponds to a quasi-static loading condition, whereas the other two loading rates are consistent with the cutting speeds observed during skin surgery^19^. Univariate hypothesis tests are performed on each of the five mechanical properties to study the statistical similarity between the two types of tissues. In addition, multivariate statistical analysis is carried out to differentiate between two types of skin tissues based on the five mechanical properties.

The paper is organized as follows. The experimental setup for the uniaxial tensile tests and the statistical analysis on the material properties are described in “Materials and methods” section. The results of the univariate and multivariate statistical analysis are presented in “Results” section along with a discussion of the results in “Discussion” section. The summary of the study is presented in “Conclusion” section, along with future directions. The description of the human skin samples and the comparison of various material models used to fit the experimental stress–strain data are given in Appendix A in supplementary information. The details of the univariate and multivariate statistical analysis are provided in Appendix B in supplementary information.

## Materials and methods

The uniaxial tensile tests are conducted on the full thickness burned ex vivo porcine skin and debrided human skin tissues at three different loading rates, that is, 0.3 mm/s, 2 mm/s, and 8 mm/s, until the rupture of the specimen. Force and displacement data collected from these experiments are used to calculate ultimate tensile stress, ultimate tensile strain, toughness, and the parameters of Veronda-Westmann hyperelastic material model^18^. These five material properties are used to compare the mechanical characteristics of porcine and human skin tissues.

The experimental protocol for the uniaxial tensile testing of full thickness burned human and porcine skin samples are presented in “Human sample testing protocol” and “Porcine sample testing protocol” sections, respectively. The calculation of the material properties from the data collected from the uniaxial tensile tests is given in “Characterization of material properties” section. The methodologies for the univariate and multivariate statistical analysis are given in “Statistical analysis” section.

### Human sample testing protocol

The human skin samples were tested at the University of Washington in Seattle, WA, and de-identified measurement data were provided for this study. The human samples were collected as debrided/discarded patient tissues that had undergone full thickness or deep partial burns, at the Harborview Medical Center Burn Unit in Seattle, Washington, in accordance with the protocol approved by the University of Washington Institutional Review Board (IRB). The informed consent requirement is waived by the IRB committee for the access and use of medical records and debrided/discarded skin tissue specimens. Deep partial burns can be classified as full thickness burns for this study as the deep partial burns can develop eschar, which requires escharotomies just as full thickness burns do. The anatomical location of the burned skin and the number of post-burn hours after the tissue had been excised were recorded, along with the demographics of each patient such as age and gender. After the burned skin tissue was excised, it was submerged in 1X phosphate-buffered saline (PBS) (pH 7.4) to prevent loss of hydration and then stored in refrigeration. The tissue sample was transferred to the University of Washington. The subcutaneous tissue was removed, and the tissue was processed into standard dog-bone shape specimens using an ASTM D638 Type V die, and the gauge length was recorded using tissue dye and a stencil. The uniaxial tensile tests were performed on a TA Instruments ElectroForce TestBench (TA Instruments, MN) instrument in a 37 °C PBS bath at loading rates of 0.3 mm/s, 2 mm/s, and 8 mm/s until failure. The loading rate of 0.3 mm/s corresponds to a quasi-static loading condition, whereas 2 mm/s and 8 mm/s are consistent with the cutting speeds observed during skin surgery^19^. The samples were preloaded to 0.10 N and the cross-sectional area was measured using laser micrometers IG-028 (Keyence, IL). Force and displacement data are collected from the testing machine. The material characterization study described above is performed in accordance with the guidelines and regulations of the IRB protocol.

For the loading rates of 0.3 mm/s, 2 mm/s, and 8 mm/s a total of 107, 105, and 108 burned skin samples excised from 14, 14, and 15 human subjects, respectively, are used in this study. The samples were obtained from 13 male and 2 female subjects in the age range 38 to 78 years. The time between the burn injury and the incision of the skin sample varies between 23 and 68 hours. In four extreme cases, skin tissues were excised after 11, 120, 139, and 360 hours. The mean thickness of the human skin samples is 2.1 ± 1.0 mm. The anatomical location of the burn injury for the 15 subjects is given in supplementary information (Appendix A).

### Porcine sample testing protocol

Locally sourced ex vivo porcine abdominal skin tissues were used in this study. The tissue was stored in refrigeration before the skin was separated from the subcutaneous and underlying muscles using a scalpel. A commercial griller (Cuisinart^®^ GR-300WS Griddler Elite Grill, Conair Corporation, NJ) was set to 450 °F, which was confirmed using an infrared camera (InfraCam, FLIR, OR), and the tissue was burned for 30 s to induce full thickness burns. This burn condition is chosen as a surrogate of burn depth, which is known to result in full thickness burn^17,20^. The tissue was submerged in 1X PBS solution until it reached room temperature, and it was then processed into standard dog-bone shape specimens using an ASTM D568 Type V die. This was done to avoid shrinkage of skin tissue during the contact heating process. The uniaxial tensile tests were performed on an Instron^®^ MTS system (INSTRON, MA) at the loading rates of 0.3 mm/s, 2 mm/s, and 8 mm/s until failure. Force and displacement data are collected from the testing machine. A total of 38, 39, and 39 samples were used for testing at three loading rates, respectively. The mean thickness of the porcine skin samples is 1.5 ± 0.7 mm.

### Characterization of material properties

**Figure 1:**
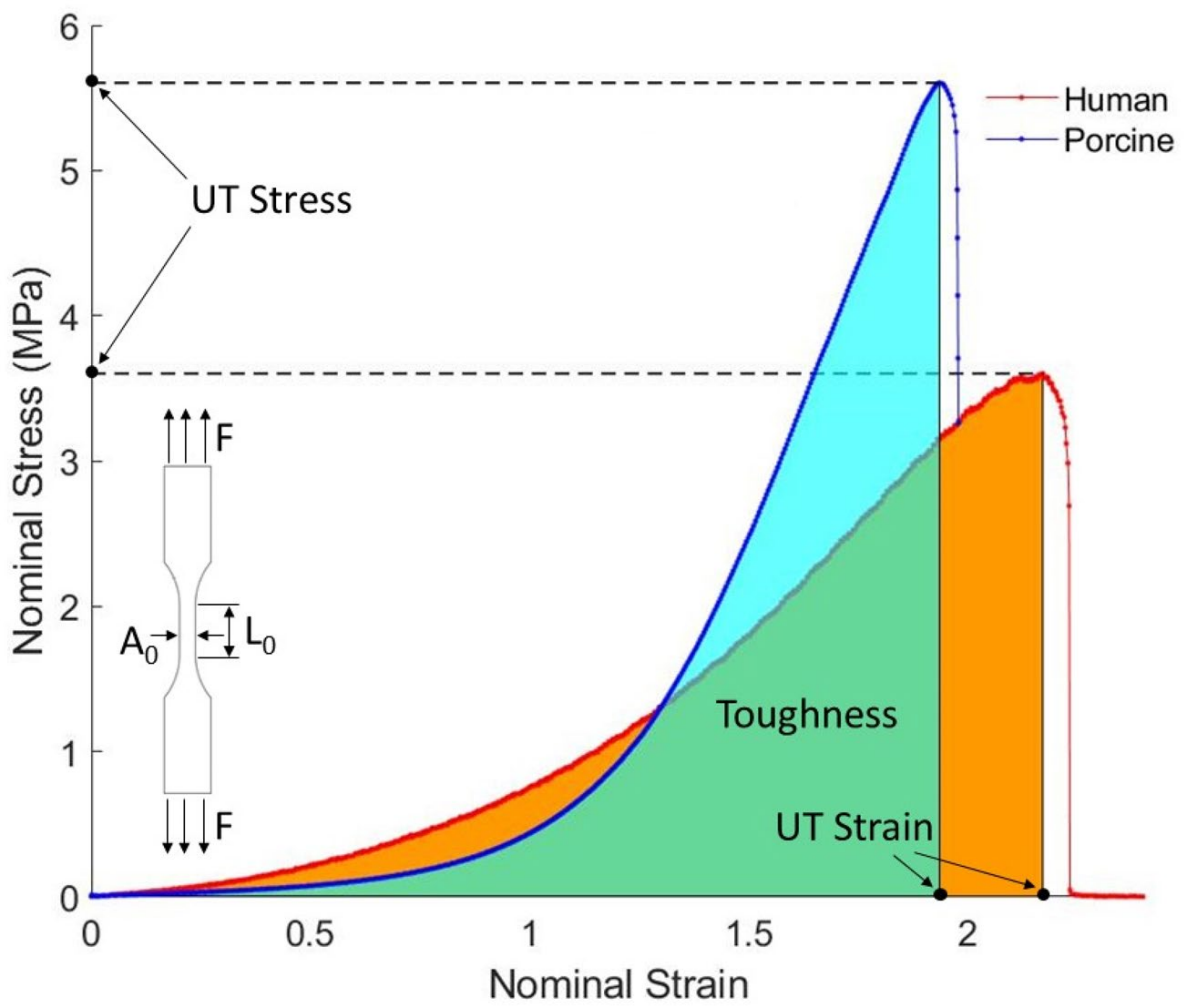
A typical nominal stress–strain curve of the dog bone skin tissue samples under the uniaxial tensile test. The applied force (F), the initial cross-section area (A_0_), and the initial length (L_0_) of the sample are shown on the dog-bone sample.

The nominal stress ${\sigma }_{N}=F/{A}_{0}$ and nominal strain ${\varepsilon }_{N}=\Delta L/{L}_{0}$ are computed using force $F$ and displacement $\Delta L$ measurements obtained from the uniaxial tensile tests of the *dog bone* samples. Here, ${A}_{0}$ is the initial cross-sectional area of the gauge section and ${L}_{0}$ is the initial length of the sample, as shown in Fig. 1. The ultimate tensile stress, ultimate tensile strain, and toughness were calculated from the nominal stress–strain curve, as shown in Fig. 1. The ultimate tensile stress describes the maximum nominal stress that the sample can withstand before it ruptures under uniaxial tensile loading. The ultimate tensile strain corresponds to the nominal strain at the ultimate tensile stress. Toughness is calculated as the area under the nominal stress–strain curve, i.e., the amount of energy per unit volume that a sample can absorb before rupturing.

The stress–strain constitutive response of the full thickness burned human and porcine skin tissues is described by the Veronda-Westmann hyperelastic material law, with the strain energy density function given as

1 $$\Psi \left({\overline{I} }_{1},{\overline{I} }_{2}\right)=\frac{\mu }{\gamma }\left({e}^{\gamma \left({\overline{I} }_{1}-3\right)}-1\right)-\mu \left({\overline{I} }_{2}-3\right),$$

where $\mu$ and $\gamma$ are two material coefficients, ${\overline{I} }_{1}$ and ${\overline{I} }_{2}$ are the first and second deviatoric strain invariants, respectively. For the uniaxial tensile test, the nominal stress for the Veronda-Westmann material is then given as

2 $${\sigma }_{NU}=2\mu \left(1-{\lambda }_{U}^{-3}\right)\left({\lambda }_{U}{e}^{\gamma \left({\overline{I} }_{1}-3\right)}-1\right),$$

where ${\lambda }_{U}=1+{\varepsilon }_{N}$ is the principle stretch along the uniaxial loading direction and ${\varepsilon }_{N}$ is the nominal strain under uniaxial loading conditions. The parameters, *μ* and *γ*, are calculated by nonlinear least squares fit of the nominal stress–strain curve to the material law. The Veronda-Westmann model was originally developed to model the behavior of ex vivo cat skin under uniaxial tensile loading^18^. It fits the experimental stress–strain curve for both the full thickness burned human and porcine skin tissues with *R*^2^ goodness-of-fit measure of 0.99 for all three loading rates. The curve fitting plots for the Veronda-Westmann model and the other hyperelastic material models typically used to model soft tissues are presented in supplementary information (Appendix A). Hence, the Veronda-Westmann model is deemed reasonable to describe the stress–strain response of full thickness burned skin tissues in this study.

### Statistical analysis

Univariate and multivariate statistical analysis is carried out on the five material properties of the full thickness burned human and porcine skin tissues, viz., ultimate tensile stress, ultimate tensile strain, toughness, and the parameters of the Veronda-Westmann hyperelastic material model, μ and γ. The methodology for the univariate and multivariate statistical analyses is presented in “Univariate statistical analysis” and “Multivariate statistical analysis”, respectively.

#### Univariate statistical analysis

The distributions of the five material properties of the full thickness burned human and porcine skin tissue samples are compared between the two types of tissues at each of the three loading rates, described in the following steps.

1. First, the Shapiro–Wilk normality test^21^ is performed on the sample data of each material property for human and porcine skin tissues and each loading rate.
2. If we can assume that a pair of sample data (human and porcine for a particular parameter and loading rate) was drawn from normal distributions:
  - The two-sample *F*-test for equal variances^22^ is carried out.
  - Depending on whether the variances of the two samples are equal or unequal, a two-sample *t*-test for equal or unequal variances^22^ is conducted, respectively.
3. If we cannot assume that a sample pair was drawn from two normal distributions,
  - the two-sample Kolmogorov–Smirnov test^23^ is performed.
  - If the two samples were drawn from continuous distributions that have the same shape, the Wilcoxon rank-sum test^22^ is performed.
  - If the two samples were not drawn from continuous distributions that have the same shape, a two-sample *t*-test for unequal variances is performed, as per the recommendation in Ruxton^24^.

The significance of all the hypothesis tests is chosen to be 0.01. A flowchart of the univariate hypothesis testing and the details of the null hypothesis for each test used in the study are defined in supplementary information (Appendix B).

For a post-hoc analysis to verify that the sample sizes exceeded the required minimum, the effect size for each sample pair is estimated by computing Cohen’s d statistic. This statistic is a numerical representation of the strength of the relationship between two variables in a population. An effect size of d = 0.2, is considered small, d = 0.5 is considered medium, and d = 0.8 is considered large^25^. The sample size estimations are calculated using G*Power 3.1.9.7^26^, with the significance level and power chosen to be 0.01 and 0.99, respectively.

#### Multivariate statistical analysis

Although univariate hypothesis testing can highlight differences in the mean or median between two classes, it cannot qualitatively describe the degree of such differences. However, this is essential since simply rejecting the null hypothesis does not offer an insight into the significance of the differences between the mean or median of the classes. Hence, a multivariate classification analysis^27^ is carried out by combining the five variables: ultimate tensile stress, ultimate tensile strain, toughness, and the parameters of the Veronda-Westmann hyperelastic material model, μ and γ.

Binary classification is carried out for each loading rate of 0.3 mm/s, 2 mm/s, and 8 mm/s based on pairwise samples (one for burned human and one for porcine specimen). The classification is performed using logistic regression^28^. A leave-one-out cross-validation is applied in order to guarantee an independent assessment of the classifiers. This is a holdout strategy, where one data point is removed from training the classifier model and later used to assess the classification accuracy of the trained model. The procedure is concluded when each data point is removed once, starting with the first and finishing with the last data point. The smaller the misclassification error, i.e., the percentage of incorrectly classified observations, the more significant is the difference between the two samples.

In addition to a binary classification based on the three different loading rates, the three datasets for human, and the three datasets for porcine are also combined. This yields two data sets that allows conducting a direct, rate independent, binary classification to assess the difference in the material properties between full thickness burned human and porcine skin tissues.

## Results

The univariate and multivariate statistical analysis of the five material properties, i.e., the ultimate tensile stress, ultimate tensile strain, toughness, and the parameters of the Veronda-Westmann material model, of full thickness burned human and porcine skin is carried out to compare the behavior of these skin types at different loading rates. The results of the univariate and multivariate analysis are presented in “Univariate statistical analysis” and “Multivariate statistical analysis” sections, respectively.

### Univariate statistical analysis

**Figure 2:**
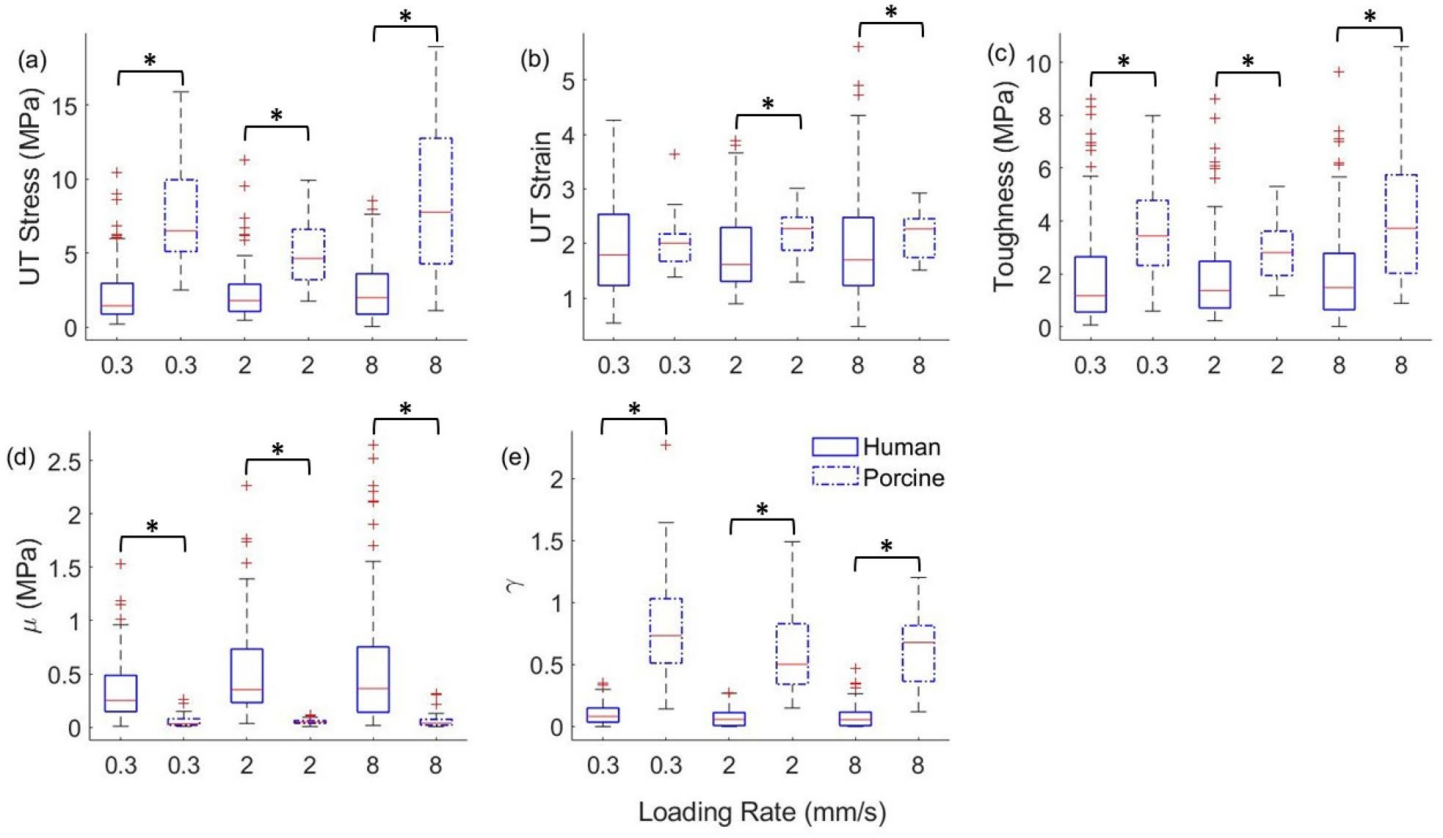
The box plots of (**a**) ultimate tensile stress (UT Stress), (**b**) ultimate tensile strain (UT Strain), (**c**) toughness, and parameters (**d**) μ and (**e**) γ of the Veronda-Westmann model for the full thickness burned human and porcine skin tissue at loading rates of 0.3 mm/s, 2.0 mm/s, and 8.0 mm/s. ‘*’ indicates a significant difference in the Wilcoxon rank-sum test or the *t*-test for unequal variances.

The samples of the full thickness burned human and porcine skin tissues with at least one of the five properties greater than three times the interquartile range of the distribution of the property were removed from the analysis. The number of samples used in the statistical analysis after removing the outliers for human and porcine skin tissues for the 0.3 mm/s, 2.0 mm/s, and 8.0 mm/s loading rates is given in Table 1. The Cohen’s d effect size for the human and porcine skin tissue samples based on the five material properties is found to be 1.4. An allocation ratio of 2.5 is chosen, which is close to that used in this study. The sample size estimation for human and porcine tissues based on the Cohen’s d value and the allocation ratio is 46 and 18, respectively. This indicates that the sample size used in this study is appropriate for the univariate hypothesis tests. The box plots of the five material properties of the human and porcine skin tissues at the three loading rates are shown in Fig. 2.

**Table 1:** The number of samples of full thickness burned human and porcine skin tissues for three loading rates after removing the outliers.

Loading rate/tissue type	0.3 mm/s	2.0 mm/s	8.0 mm/s
Human skin	95	92	102
Porcine skin	38	39	38

The results of the univariate hypothesis tests are detailed in supplementary information (Appendix B). The hypothesis tests show that the means or medians of ultimate tensile stress, toughness, and the two parameters, μ and γ, of the Veronda-Westmann model are not equal for all loading rates between the human and porcine skin tissue samples. The difference in the median and variance of the properties between the human and porcine skin tissues at all loading rates is also observed in Fig. 2. Based on the Kolmogorov–Smirnov test, we reject the claim that the shape of the two distributions is identical for the two tissues for the ultimate tensile stress at loading rate of 8 mm/s and for the two parameters, μ and γ of the Veronda-Westmann model at all three loading rates. Although, the median of the ultimate tensile strain of human and porcine skin tissues is equal for the loading rate of 0.3 mm/s based on the Wilcoxon rank-sum test, it is not equal for the loading rates of 2 mm/s and 8 mm/s.

### Multivariate statistical analysis

The multivariate analysis using logistic regression statistical model is carried out to differentiate between full thickness burned human and porcine skin tissues based on the five material properties, namely, the ultimate tensile stress, the ultimate tensile strain, toughness, and the parameters μ and γ of the Veronda-Westmann material law. Binary classification is performed to separate human and porcine skin tissues for each of the loading rates, i.e., 0.3 mm/s, 2 mm/s, and 8 mm/s. In addition, human and porcine skin samples are classified by combining samples from all three loading rates into respective classes. Table 2 summarizes the performance metrics, such as traditional accuracy, sensitivity, specificity, F1-score, area under the receiver operating characteristic curve (ROC-AUC), as well as Matthew’s correlation coefficient (MCC)^29^, Fowlkes-Mallow’s index (FMI)^30^, and Adjusted Rand index (ARI)^31^, computed from the confusion matrix obtained from the leave-one-out cross-validation. The confusion matrices for the binary classifications are provided in supplementary information (Appendix B).

**Table 2:** Performance metrics of the classifier obtained from leave-one-out cross-validation.

Performance metrics/loading rates	Accuracy	Sensitivity	Specificity	F1-score	ROC-AUC	MCC	FMI	ARI
0.3 mm/s	0.9699	0.9789	0.9474	0.9789	0.9632	0.9263	0.9501	0.8786
2.0 mm/s	0.9771	0.9783	0.9744	0.9836	0.9763	0.9458	0.9608	0.9077
8.0 mm/s	0.9500	0.9608	0.9211	0.9655	0.9409	0.8748	0.9201	0.8009
All loading rates combined	0.9653	0.9792	0.9304	0.9759	0.9548	0.9145	0.9435	0.8610

**Figure 3:**
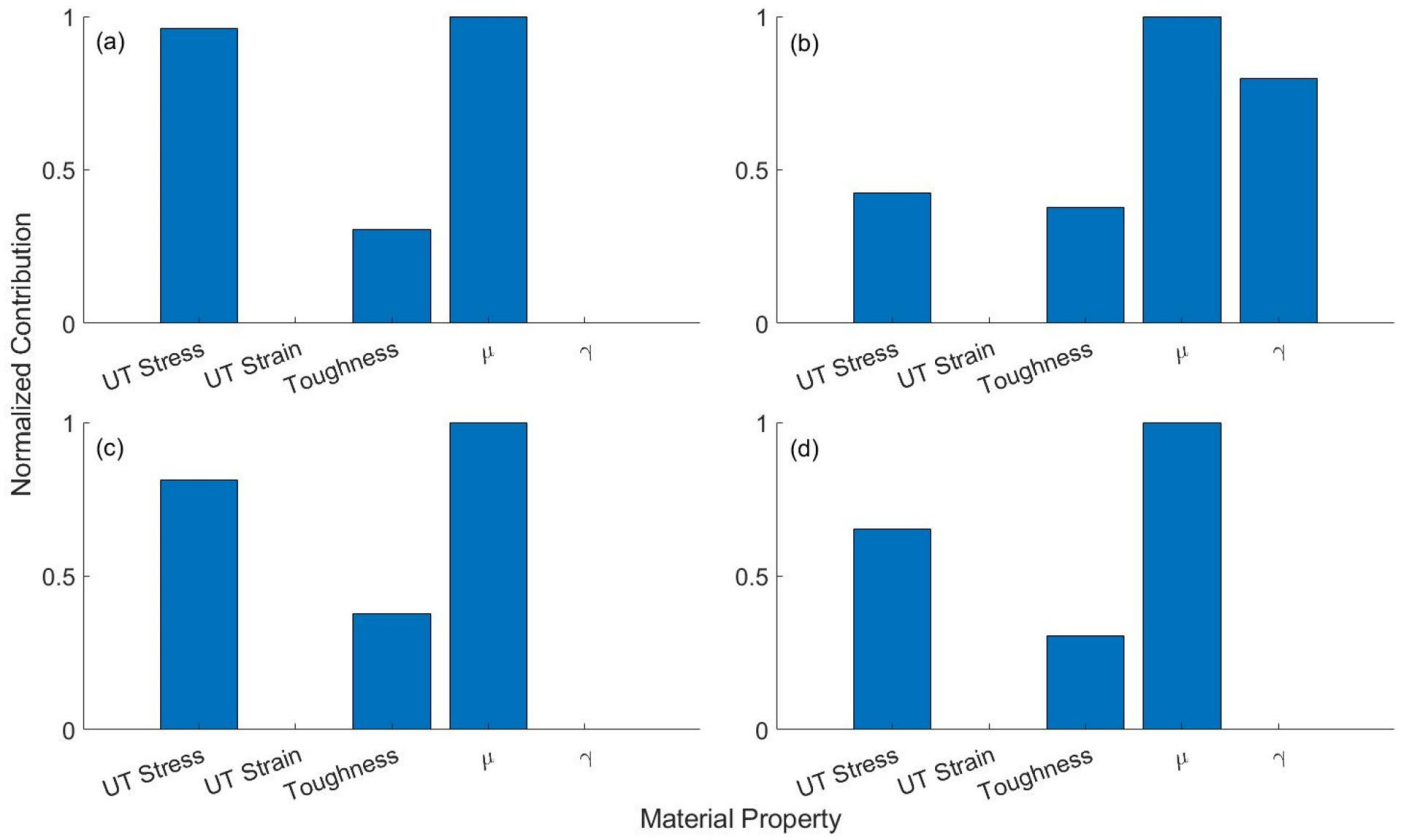
Contribution of each of the five material properties in the binary classification of full thickness burned human and porcine skin tissue samples under loading rate of (**a**) 0.3 mm/s, (**b**) 2 mm/s, (**c**) 8 mm/s, (**d**) all three loading rates combined.

The human and porcine skin types can be separated with a high degree of accuracy (> 0.95), sensitivity (> 0.96), and specificity (> 0.92) for all loading rates (Table 2). This can also be confirmed by the high value of the F1-score, as well as the MCC, FMI, and ARI metrics further indicates that the two tissue types can be accurately classified into their respective classes based on five mechanical properties, irrespective of loading conditions. A factor analysis of the classifier is finally performed to quantify the contribution of each mechanical property in differentiating between the two types of skin tissues. This is to examine how influential each of the five material properties is in differentiating between the two skin types. Figure 3 shows that the parameter *μ* of the material model and the ultimate tensile strain, respectively, are the most and the least discriminative factors among the five properties for all loading conditions. The material model parameter γ only contributes to differentiating the two skin types for loading rate 2 mm/s.

## Discussion

The univariate and multivariate statistical analyses using five mechanical properties, *i*.*e*., ultimate tensile stress, ultimate tensile strain, toughness, and two parameters of the Veronda-Westmann material model, reveal that the mechanical characteristic of full thickness burned human skin is significantly different from that of porcine skin tissues, irrespective of the three loading conditions tested in this study. These findings are consistent with previous studies that indicate that the tangent modulus of human skin is different from the porcine skin tissues, even at similar anatomical locations^13^. However, it contradicts the assumption that porcine skin is a de facto surrogate for human skin to evaluate the mechanical characteristics^3,4,9^.

The difference in the mechanical behavior of the full thickness burned human and porcine skin tissues can be attributed to the structural, compositional, and functional differences between these tissue types. The elastic fiber content in dermis is different between the two types of skin tissue^8^. Phenomenological models further show that the elastic modulus of the skin of animals and humans is a function of the thickness of each skin layer, which are not similar between humans and porcine^32^. In addition, the volume fraction of moisture content in healthy porcine skin tissue is more than that of human skin. The corneocytes of the stratum corneum retain moisture when skin tissue is not hydrated, while the corneocytes and lipids in the stratum corneum swell with hydration and thereby significantly alter the tangent modulus of the tissue. These layers of corneocyte and lipid content are different between human and porcine tissues^15^. The hydrogen bonding states of water and the natural moisturizing factor concentration is also lower in porcine skin compared to human skin which affects the permeability of the two skin types^33^. The natural moisturizing factor is composed of water-soluble compounds such as free amino acids and its derivatives, inorganic salts, sugars, lactic acid, urea etc.^34^. Further, the water loss in a healthy pig is mainly through rapid evaporation and it is found to be similar in in vivo and ex vivo porcine skin tissues. Whereas the water loss in human skin at higher temperatures is significantly more than that in porcine skin^35^. Hence, it is expected that the water content in a full thickness burned porcine skin is significantly different from that of burned human skin. The viscoelastic behavior of skin tissue depends on the interaction between collagen, proteoglycans, and water molecules^4^. A difference in the water content between the two types of burned skin severely affects this interaction among the constituents of skin tissue which results in the dissimilar mechanical properties.

Further, the lipid content and keratin type and its structure in the stratum corneum of the unburned skin tissue is different between human and porcine^33^. It is observed that the disruption of the lipid and keratin structures in the stratum corneum causes significant changes to the permeability and mechanical response of burned skin tissue^36^. Hence, it is expected that the difference in the lipids and keratin content and structure between burned human and porcine skin tissue may lead to a significant difference in mechanical properties, as observed in this study.

The effect of the anatomical location of the full thickness burned skin tissue on the mechanical properties could not be studied due to the insufficient human skin tissue samples available for different anatomical locations. It is unlikely that the difference in mechanical characteristics of human and porcine skin stems from the difference in the experimental conditions and anatomical location of the tissue samples. The mechanical properties of skin tissues are known to change with temperature due to collagen denaturation^4^. Collagen and elastin content contribute to the elasticity of the skin tissue^1^. Collagen fiber orientation is responsible for the variation in elasticity and strength of the skin tissue along different orientations at various anatomical locations of the skin^13^. However, when the skin tissue undergoes full thickness burn, the collagen fibers undergo denaturation^37^. The fibers transform from an organized crystalline structure to a random gel-like state^38^. Hence, the difference in burn temperature and collagen fiber orientation in samples from anatomically different locations is unlikely to contribute to the statistically significant difference in the mechanical properties of the two skin tissue types.

In the study, in vivo for human skin samples are used, whereas it is ex vivo for the porcine skin samples. However, both human and porcine skin tissue samples have undergone full thickness burn. For the human skin tissue, the burn condition is confirmed by experienced burn care personnel at the Harborview Medical Center Burn Unit, Seattle, WA. For the porcine skin tissue, the contact burn at 450 °F and for 30 s is known to cause full thickness burn^17,20^. Hence, we expect similar burn-related thermal damage in both tissue types. Similar comparisons of in vivo human skin and ex vivo porcine skin have been studied in the literature^33^. It should also be noted that mechanical properties of ex vivo porcine skin at high temperatures are studied in literature to understand the mechanical behavior of human skin at high temperatures^4^.

The conclusions of the present study are limited to the mechanical characteristics of full thickness burned human and porcine skin. Further research needs to be conducted to explore the differences between human and porcine tissues for other properties under thermal damage.

## Conclusion

Uniaxial tensile tests are conducted on full thickness burned ex vivo human and porcine skin tissues at three loading rates of 0.3 mm/s relating to quasi-static loading, 2 mm/s, and 8 mm/s relating to the rates in skin surgery. The ultimate tensile stress, ultimate tensile strain, toughness, and the two parameters of the Veronda-Westmann material model are calculated for the human and porcine samples at the three loading rates using the force–displacement data from the tensile tests. Univariate hypothesis tests indicate a significant difference in all five material properties between the two types of skin tissues for all loading rates. The classification accuracy from the multivariate analysis between the human and porcine skin tissues is more than 95% when considering samples from each loading rate separately and together using logistic regression statistical model. Hence, the full thickness burned porcine skin tissue is statistically not similar to that of the human skin tissue. This observation is significant for the development of training protocols and high-fidelity simulators to train burn care providers. This study could be further extended to investigate the effect of loading rates on the rate-dependent viscoelastic response of the severely burned skin tissues.

## Supplementary Information

Supplementary Information.

## Data Availability

The datasets generated during and/or analyzed during the current study are available from the corresponding author on reasonable request.

## References

[1] Summerfield A, Meurens F, Ricklin ME (2015). The immunology of the porcine skin and its value as a model for human skin. Mol. Immunol..

[2] Sullivan TP, Eaglstein WH, Davis SC, Mertz P (2001). The pig as a model for human wound healing. Wound Repair Regen..

[3] Shergold OA, Fleck NA, Radford D (2006). The uniaxial stress versus strain response of pig skin and silicone rubber at low and high strain rates. Int. J. Impact Eng..

[4] Zhou B, Xu F, Chen CQ, Lu TJ (2010). Strain rate sensitivity of skin tissue under thermomechanical loading. Philos. Trans. R. Soc. A.

[5] Abdullahi A, Amini-Nik S, Jeschke MG (2014). Animal models in burn research. Cell. Mol. Life Sci..

[6] Arnoczky SP, Aksan A (2000). Thermal modification of connective tissues: Basic science considerations and clinical implications. J. Am. Acad. Orthop. Surg..

[7] Clark JA, Cheng JCY, Leung KS, Leung PC (1987). Mechanical characterisation of human postburn hypertrophic skin during pressure therapy. J. Biomech..

[8] Marcarian HQ, Calhoun ML (1966). Microscopic anatomy of the integument of adult swine. Am. J. Vet. Res..

[9] Lim J, Hong J, Chen WW, Weerasooriya T (2011). Mechanical response of pig skin under dynamic tensile loading. Int. J. Impact Eng..

[10] Jansen LH, Rottier PB (1958). Some mechanical properties of human abdominal skin measured on excised strips. Dermatology.

[11] Wong VW, Levi K, Akaishi S, Schultz G, Dauskardt RH (2012). Scar zones: Region-specific differences in skin tension may determine incisional scar formation. Plast. Reconstr. Surg..

[12] Ni Annaidh A, Bruyère K, Destrade M, Gilchrist MD, Otténio M (2012). Characterization of the anisotropic mechanical properties of excised human skin. J. Mech. Behav. Biomed. Mater..

[13] Pissarenko A, Meyers MA (2020). The materials science of skin: Analysis, characterization, and modeling. Prog. Mater. Sci..

[14] Ranamukhaarachchi SA (2016). Development and validation of an artificial mechanical skin model for the study of interactions between skin and microneedles. Macromol. Mater. Eng..

[15] Ranamukhaarachchi SA (2016). A micromechanical comparison of human and porcine skin before and after preservation by freezing for medical device development. Sci. Rep..

[16] Dunn MG, Silver FH, Swann DA (1985). Mechanical analysis of hypertrophic scar tissue: Structural basis for apparent increased rigidity. J. Investig. Dermatol..

[17] Ye H, Rahul, Dargar S, Kruger U, De S (2018). Ultrasound elastography reliably identifies altered mechanical properties of burned soft tissues. Burns.

[18] Veronda DR, Westmann RA (1970). Mechanical characterization of skin—finite deformations. J. Biomech..

[19] Loh SA (2009). Comparative healing of surgical incisions created by the PEAK PlasmaBlade, conventional electrosurgery, and a scalpel. Plast. Reconstr. Surg..

[20] Branski LK, Mittermayr R, Herndon DN, Norbury WB, Masters OE, Hofmann M, Traber DL, Redl H, Jeschke MG (2008). A porcine model of full-thickness burn, excision and skin autografting. Burns.

[21] Shapiro SS, Wilk MB (1965). An analysis of variance test for normality (complete samples). Biometrika.

[22] Montgomery DC, Runger GC (2011). Applied Statistics and Probability for Engineers.

[23] Massey FJ (1951). The Kolmogorov–Smirnov test for goodness of fit. J. Am. Stat. Assoc..

[24] Ruxton GD (2006). The unequal variance t-test is an underused alternative to Student's t-test and the Mann–Whitney U test. Behav. Ecol..

[25] Cohen J (1988). Statistical Power Analysis for the Behavioral Sciences.

[26] Faul F, Erdfelder E, Buchner A, Lang AG (2009). Statistical power analyses using G* Power 3.1: Tests for correlation and regression analyses. Behav. Res. Methods.

[27] Kent JT, Bibby J, Mardia KV (1995). Multivariate Analysis.

[28] Hosmer DW, Lemeshow S (2000). Applied Logistic Regression.

[29] Matthews BW (1975). Comparison of the predicted and observed secondary structure of T4 phage lysozyme. Biochim. Biophys. Acta Protein Struct..

[30] Fowlkes EB, Mallows CL (1983). A method for comparing two hierarchical clusterings. J. Am. Stat. Assoc..

[31] Hubert L, Arabie P (1985). Comparing partitions. J. Classif..

[32] Wei JC (2017). Allometric scaling of skin thickness, elasticity, viscoelasticity to mass for micro-medical device translation: From mice, rats, rabbits, pigs to humans. Sci. Rep..

[33] Choe C, Schleusener J, Lademann J, Darvin ME (2018). Human skin in vivo has a higher skin barrier function than porcine skin ex vivo—comprehensive Raman microscopic study of the stratum corneum. J. Biophotonics.

[34] Fowler J (2012). Understanding the role of natural moisturizing factor in skin hydration. Pract. Dermatol..

[35] Moritz AR, Henriques FC (1947). Studies of thermal injury: II. The relative importance of time and surface temperature in the causation of cutaneous burns. Am. J. Pathol..

[36] Park JH, Lee JW, Kim YC, Prausnitz MR (2008). The effect of heat on skin permeability. Int. J. Pharm..

[37] Moritz AR (1947). Studies of thermal injury: III. The pathology and pathogenesis of cutaneous burns. An experimental study. Am. J. Pathol..

[38] Flory PJ, Garrett RR (1958). Phase transitions in collagen and gelatin systems. J. Am. Chem. Soc..

