# Supplementary material for: Thermally damaged porcine skin is not a surrogate mechanical model of human skin
Source: Sci Rep. 2022 Mar 16;12:4565. doi: 10.1038/s41598-022-08551-z (PMC8927453; doi:10.1038/s41598-022-08551-z)
Supplement: Supplementary file 1 — Supplementary Information. [file 41598_2022_8551_MOESM1_ESM.docx]

**Supplementary Information**

**Thermally damaged porcine skin is not a surrogate mechanical model of human skin**

Samara Gallagher,^1,2^ Uwe Kruger,^2,3^ Kartik Josyula,^2^ Rahul*,^2^ Alex Gong,^4^ Agnes Song,^4^ Robert Sweet,^4^ Basiel Makled,^5^ Conner Parsey,^5^ Jack Norfleet,^5^ Suvranu De,^1,2,3^

^1^ Department of Mechanical, Aerospace, and Nuclear Engineering

^2^ Center for Modeling, Simulation, and Imaging in Medicine

^3^ Department of Biomedical Engineering

Rensselaer Polytechnic Institute, Troy NY

^4^ Center for Research in Education and Simulation Technologies, University of Washington, Seattle WA

^5^ U.S. Army Combat Capabilities Development Command - Soldier Center, Simulation and Training Technology Center, Orlando FL

**Appendix A**

**A.1. Anatomical location of human skin samples**

The anatomical location of the debrided/discarded full thickness (or deep partial-thickness) burned human skin tissues for 15 subjects is given in Table A.1. The number of dog bone samples collected from each subject is also given in Table A.1.

Table A.1. Anatomical location of the burn injury of the human subjects.

| **Subject No.** | **Anatomical location of burn injury** | **Number of samples** | | |
| --- | --- | --- | --- | --- |
|  |  | **0.3 mm/s** | **2 mm/s** | **8 mm/s** |
| 1 | Right upper extremity | 1 | 1 | 1 |
| 2 | Right arm | 0 | 0 | 2 |
| 3 | Left flank and Left arm | 9 | 9 | 11 |
| 4 | Left and right thighs | 8 | 9 | 9 |
| 5 | Left and right arms and Chest | 10 | 9 | 9 |
| 6 | Flank | 2 | 2 | 2 |
| 7 | Right forearm | 2 | 2 | 1 |
| 8 | Abdomen | 3 | 3 | 3 |
| 9 | Chest | 4 | 3 | 4 |
| 10 | Left thigh (posterior) | 7 | 7 | 7 |
| 11 | Bilateral calf, thigh, arm, and flank | 34 | 33 | 32 |
| 12 | Right arm, flank, and thigh | 10 | 10 | 10 |
| 13 | Right thigh to toes, Left shin to toes | 8 | 8 | 8 |
| 14 | Right flank and shoulder (thorax) | 7 | 7 | 7 |
| 15 | Right buttock | 2 | 2 | 2 |

**A.2. Comparison of hyperelastic material laws**

The Veronda-Westmann hyperelastic material [1] model is compared with the Arruda-Boyce material model [2] and the reduced second order polynomial model [3]. These material models are fit to the experimental nominal stress-strain data using the least squares method. A typical curve fit for the three models is shown in Figure A.1 for each of human and porcine skin tissue samples and for each loading rate, i.e., 0.3 mm/s, 2 mm/s, and 8 mm/s. The Veronda-Westmann material model has the best fit to the data for both types of skin tissues with the R^2^ goodness-of-fit measure of 0.99 for all three loading rates. Hence, the Veronda-Westmann model is used in the present study to describe the stress-strain response of the full thickness burned human and porcine skin tissues at various loading rates.


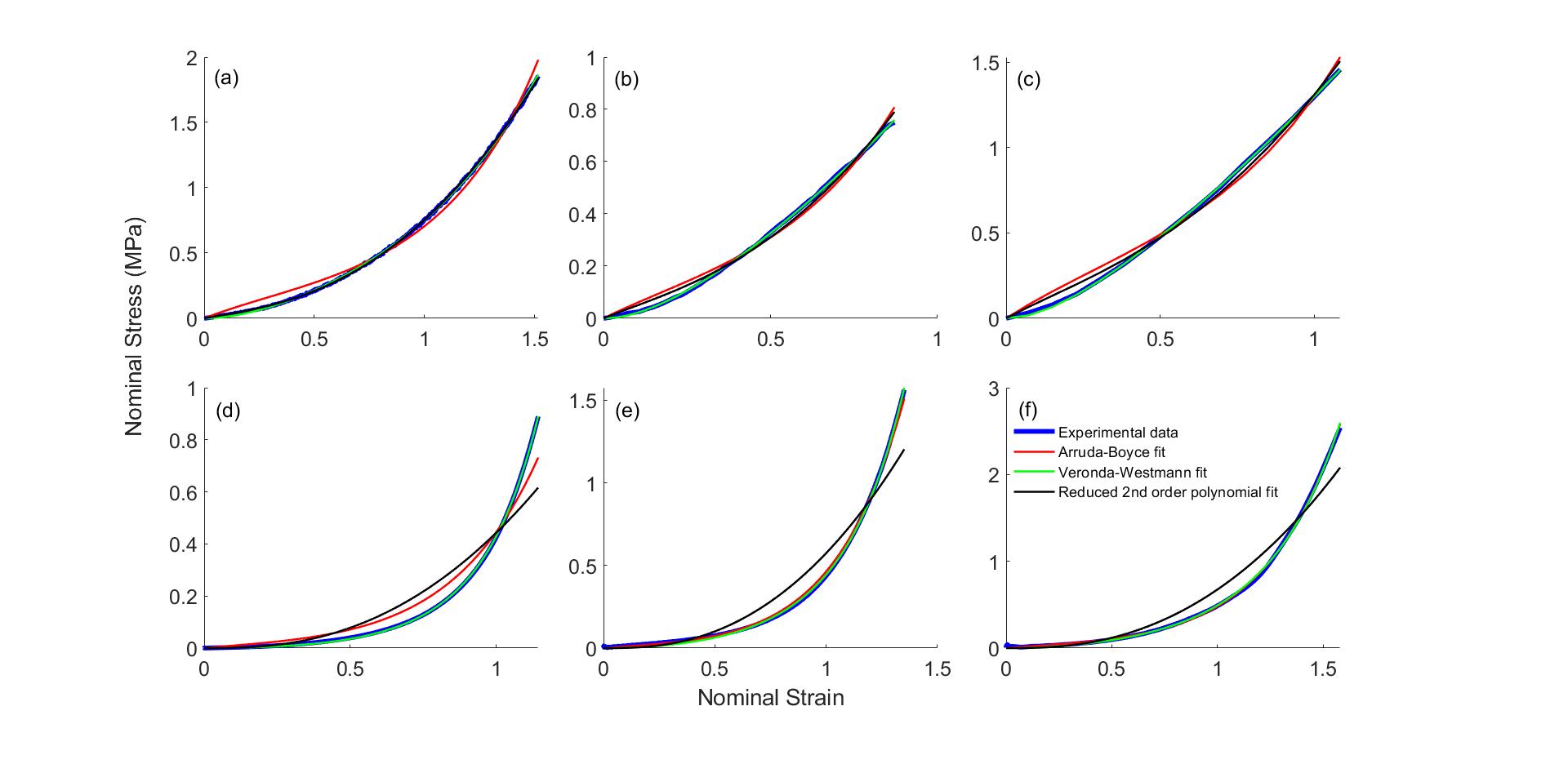


Figure A.1. Curve-fitting of the Arruda-Boyce model, reduced second order polynomial model, and the Veronda-Westmann model to the experimental nominal stress-strain data from the uniaxial tensile tests on full thickness burned human tissue loaded at (a) 0.3 mm/s, (b) 2 mm/s, (c) 8 mm/s, and full thickness burned porcine tissue loaded at (d) 0.3 mm/s, (e) 2 mm/s, (f) 8 mm/s.

**Appendix B**

**B.1. Univariate hypothesis tests**

A flowchart of the univariate hypothesis testing is given in Figure B.1.


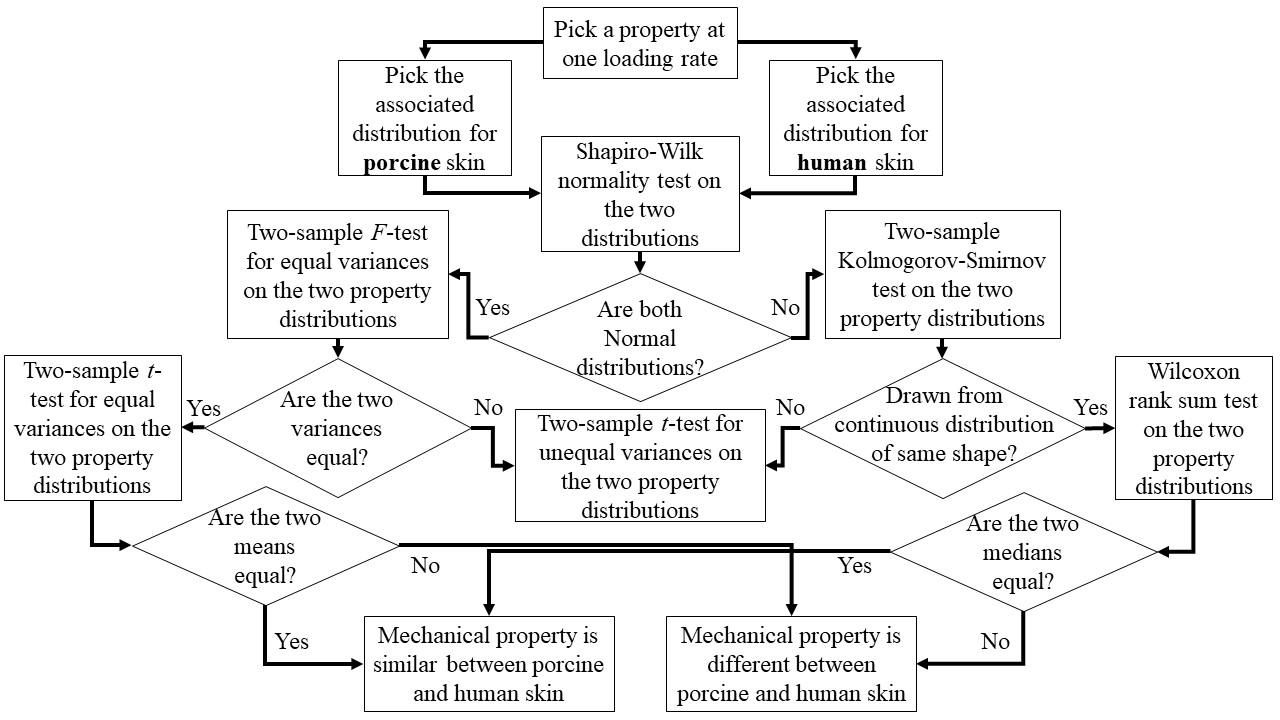


Figure B.1. The univariate hypothesis test flowchart to compare each of the five material property distributions between full thickness burned human and porcine skin at each loading rate.

The null hypotheses for all the hypothesis tests used in the present study are presented. The alternate hypothesis for each test is the opposite of the null hypothesis defined for the test.

The null hypothesis for the Shapiro-Wilk normality test is *H*_0_: *the data was drawn from a normal distribution*. The null hypothesis for the two-sample *F*-test for equal variances is *H*_0_: *the data sets were drawn from normal distributions with the same variance*. The null hypothesis for the two-sample *t*-test for equal variances is *H*_0_: *the data sets were drawn independently from two normal distributions with equal means but unknown and equal variances*. The null hypothesis for the two-sample *t*-test for unequal variances is *H*_0_: *the two random samples were drawn independently from normal distributions with equal means but unknown and unequal variances*.

The null hypothesis for the two-sample Kolmogorov-Smirnov test is *H*_0_: *the random samples were independently drawn from distributions that have the same shape but may have a different mean*. The null hypothesis for the Wilcoxon rank-sum test is *H*_0_: *the two random samples were drawn from continuous distributions with the same shape and equal medians*.

The results from the Shapiro-Wilk normality test of the five material properties at the three loading rates for human and porcine skin tissues are given in Table B.1. The univariate statistical analysis of the comparison of the material properties between different loading rates between the full thickness burned human and porcine skin tissues is given Table B.2.

Table B.1. The *p*-value of the Shapiro-Wilk normality test of the five material properties of human and porcine skin tissues at three loading rates.

| **Material Property** | **Loading Rate** | **Human** | | **Porcine** | |
| --- | --- | --- | --- | --- | --- |
|  |  | ***p*-Value** | **Normal Distribution?** | ***p*-Value** | **Normal Distribution?** |
| UT Stress | 0.3 mm/s | 0.0 | No | 0.0333 | Yes |
|  | 2 mm/s | 0.0 | No | 0.1101 | Yes |
|  | 8 mm/s | 0.0 | No | 0.0192 | Yes |
| UT Strain | 0.3 mm/s | 0.0001 | No | 0.0041 | No |
|  | 2 mm/s | 0.0 | No | 0.5603 | Yes |
|  | 8 mm/s | 0.0 | No | 0.0222 | Yes |
| Toughness | 0.3 mm/s | 0.0 | No | 0.0312 | Yes |
|  | 2 mm/s | 0.0 | No | 0.1427 | Yes |
|  | 8 mm/s | 0.0 | No | 0.0383 | Yes |
| μ | 0.3 mm/s | 0.0 | No | 0.0 | No |
|  | 2 mm/s | 0.0 | No | 0.1399 | Yes |
|  | 8 mm/s | 0.0 | No | 0.0 | No |
| γ | 0.3 mm/s | 0.0 | No | 0.0058 | No |
|  | 2 mm/s | 0.0 | No | 0.0199 | Yes |
|  | 8 mm/s | 0.0 | No | 0.1748 | Yes |

Table B.2. The *p*-value of univariate statistical tests to compare the five material properties between human and porcine skin tissues at three loading rates.

| **Material Property** | **Loading Rate** | **Statistical Test** | ***p* Value** | **Conclusion** |
| --- | --- | --- | --- | --- |
| UT Stress | 0.3 mm/s | Kolmogorov-Smirnov |  | Same distribution |
|  |  | Wilcoxon rank-sum | 0.0 | Unequal medians |
|  | 2 mm/s | Kolmogorov-Smirnov |  | Same distribution |
|  |  | Wilcoxon rank-sum | 0.0 | Unequal medians |
|  | 8 mm/s | Kolmogorov-Smirnov |  | Different distributions |
|  |  | Two sample t test | 0.0 | Unequal medians |
| UT Strain | 0.3 mm/s | Kolmogorov-Smirnov |  | Same distribution |
|  |  | Wilcoxon rank-sum | 0.1979 | Equal medians |
|  | 2 mm/s | Kolmogorov-Smirnov |  | Same distribution |
|  |  | Wilcoxon rank-sum | 0.0009 | Unequal medians |
|  | 8 mm/s | Kolmogorov-Smirnov |  | Same distribution |
|  |  | Wilcoxon rank-sum | 0.0073 | Unequal medians |
| Toughness | 0.3 mm/s | Kolmogorov-Smirnov |  | Same distribution |
|  |  | Wilcoxon rank-sum | 0.0 | Unequal medians |
|  | 2 mm/s | Kolmogorov-Smirnov |  | Same distribution |
|  |  | Wilcoxon rank-sum | 0.0 | Unequal medians |
|  | 8 mm/s | Kolmogorov-Smirnov |  | Same distribution |
|  |  | Wilcoxon rank-sum | 0.0 | Unequal medians |
| μ | 0.3 mm/s | Kolmogorov-Smirnov |  | Different distributions |
|  |  | Two sample t test | 0.0 | Unequal medians |
|  | 2 mm/s | Kolmogorov-Smirnov |  | Different distributions |
|  |  | Two sample t test | 0.0 | Unequal medians |
|  | 8 mm/s | Kolmogorov-Smirnov |  | Different distributions |
|  |  | Two sample t test | 0.0 | Unequal medians |
| γ | 0.3 mm/s | Kolmogorov-Smirnov |  | Different distributions |
|  |  | Two sample t test | 0.0 | Unequal medians |
|  | 2 mm/s | Kolmogorov-Smirnov |  | Different distributions |
|  |  | Two sample t test | 0.0 | Unequal medians |
|  | 8 mm/s | Kolmogorov-Smirnov |  | Different distributions |
|  |  | Two sample t test | 0.0 | Unequal medians |

**B.2. Multivariate statistical analysis**

The confusion matrices obtained from leave-one-out cross-validation using logistic regression statistical model are provided in Table B.3.

Table B.3. Confusion matrix for binary classification of full thickness burned human and porcine skin tissues for each loading rate and for all the loading rates using logistic regression

| **Loading Rate: 0.3 mm/s** | | |  | **Loading Rate: 2 mm/s** | | |
| --- | --- | --- | --- | --- | --- | --- |
|  | **Human** | **Porcine** |  |  | **Human** | **Porcine** |
| **Human** | 93 | 2 |  | **Human** | 90 | 2 |
| **Porcine** | 2 | 36 |  | **Porcine** | 1 | 38 |
|  |  |  |  |  |  |  |
| **Loading Rate: 8 mm/s** | | |  | **All Loading Rates** | | |
|  | **Human** | **Porcine** |  |  | **Human** | **Porcine** |
| **Human** | 98 | 4 |  | **Human** | 283 | 6 |
| **Porcine** | 3 | 35 |  | **Porcine** | 8 | 107 |

**References**

1. Veronda, D. R. & Westmann, R. A. Mechanical characterization of skin—finite deformations. *J. Biomech.* **3**, 111-124 (1970).
2. Arruda, E. M. & Boyce, M. C. A three-dimensional constitutive model for the large stretch behavior of rubber elastic materials. *J. Mech. Phys. Solids* **41**, 389-412 (1993).
3. Rivlin, R. S. & Saunders, D. W. Large elastic deformations of isotropic materials VII. Experiments on the deformation of rubber. *Philos. Trans. Royal Soc. A* **243**, 251-288 (1951).
